# The Roles of Membrane Technology in Artificial Organs: Current Challenges and Perspectives

**DOI:** 10.3390/membranes11040239

**Published:** 2021-03-28

**Authors:** Bao Tran Duy Nguyen, Hai Yen Nguyen Thi, Bich Phuong Nguyen Thi, Dong-Ku Kang, Jeong F. Kim

**Affiliations:** 1 Department of Energy and Chemical Engineering, Incheon National University, Incheon 22012, Korea; bao.nguyen@inu.ac.kr (B.T.D.N.); haiyen0107@inu.ac.kr (H.Y.N.T.); 202022027@inu.ac.kr (B.P.N.T.); 2 Department of Chemistry, Incheon National University, Incheon 22012, Korea; 3 Innovation Center for Chemical Engineering, Incheon National University, Incheon 22012, Korea

**Keywords:** membrane, artificial organs, artificial kidney, artificial lung, artificial liver, bioartificial pancreas, biocompatibility

## Abstract

The recent outbreak of the COVID-19 pandemic in 2020 reasserted the necessity of artificial lung membrane technology to treat patients with acute lung failure. In addition, the aging world population inevitably leads to higher demand for better artificial organ (AO) devices. Membrane technology is the central component in many of the AO devices including lung, kidney, liver and pancreas. Although AO technology has improved significantly in the past few decades, the quality of life of organ failure patients is still poor and the technology must be improved further. Most of the current AO literature focuses on the treatment and the clinical use of AO, while the research on the membrane development aspect of AO is relatively scarce. One of the speculated reasons is the wide interdisciplinary spectrum of AO technology, ranging from biotechnology to polymer chemistry and process engineering. In this review, in order to facilitate the membrane aspects of the AO research, the roles of membrane technology in the AO devices, along with the current challenges, are summarized. This review shows that there is a clear need for better membranes in terms of biocompatibility, permselectivity, module design, and process configuration.

## 1. Introduction

Every living organism strives to maintain homeostasis with the surrounding environments. The cells, tissues, and organs of a living organism control its internal environments to maintain safe concentration ranges in order to maintain life. In cases of unexpected tissue damage or organ failures, cells have an extraordinary ability to repair themselves and to regain homeostasis. However, some acute and chronic damages can be irreversible, leading to life-threatening failures that require medical intervention and treatment. 

**Figure 1:**
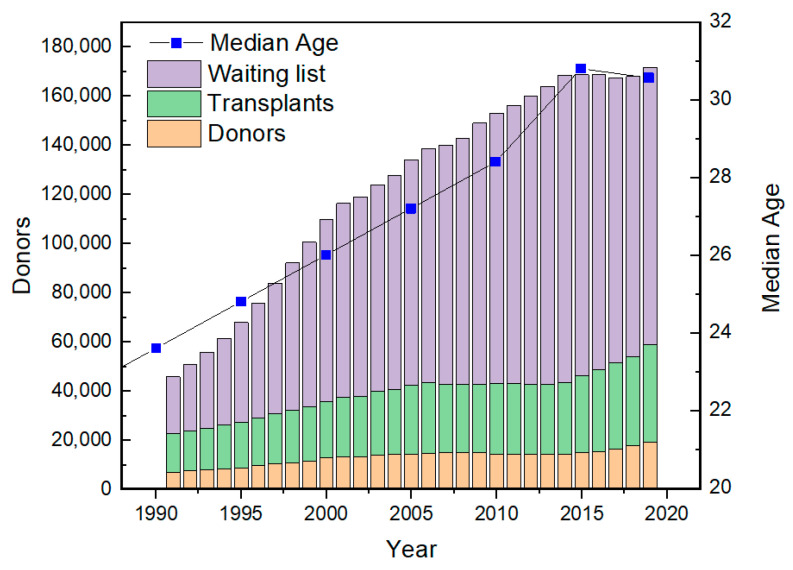
Number of people receiving transplants, waiting for transplants, and donors in the US (data obtained from [5]), plotted together with the statistic of the World Median Age (data obtained from [6]).

The median age of the world population has increased steadily (Figure 1). Such an aging population inevitably leads to higher chances of organ failures, and the best available treatment for such patients now is organ transplantation. However, as shown in Figure 1, the gap between the number of organ donors and the number of patients has expanded steadily and is only expected to get wider. In addition, although the stem-cell-derived treatment could be a promising option in the future [1,2,3,4], it has not been fully developed yet. Hence, currently, the optimal solution for organ failure patients in the short-term is artificial organ (AO) technology, until the patient recovers (i.e., bridge-to-recovery) or until the patient receives an organ transplant (i.e., bridge-to-transplantation). 

The advancements in AO technology are truly astonishing, and they are increasingly becoming important, even necessary, in the modern medical treatments. Nowadays, AO technology can partly augment the functions of the human organs by maintaining physiochemical gradients in the safe range. Many of these AOs employ synthetic polymeric membranes to assist the physical and chemical functions of the failed organs. Particularly, AOs that employ membrane technology includes artificial lung, kidney, liver, and pancreas. 

Membrane technology has also made incredible strides in the past few decades. Notably, water purification and desalination membranes have reached the state-of-the-art, dominating more than half of the desalination market [7]. Ion exchange membranes such as Nafion have become a main component for fuel cell technology. Moreover, gas separation membranes are now a key player in many fields including CO_2_ capture and hydrogen purification [8]. As a separate front, membranes for the healthcare and biomedical markets, which are the main topic of this review, have steadily increased their market share [9]. 

The market size of the artificial kidney (e.g., hemodialysis) reached USD 74 billion in 2019 [10]. As for the artificial lung market, also commonly known as ECMO (extracorporeal membrane oxygenator), is expected to grow up to USD 305 million by 2021 [11]. However, this forecast may have changed with the recent outbreak of COVID-19 in 2020, as ECMO is one of the key treatments for severe COVID-19 patients. The artificial liver and pancreas markets are still relatively small because the technology is not mature enough and is as yet without firm clinical effectiveness data. 

Surprisingly, despite the considerable market size of the healthcare industries, the literature on membrane research in such fields is relatively scarce compared to other applications such as water and gas separation membranes. In fact, there is a large amount of literature that reports the use of AO for clinical research, but not as many that actually develop AO membranes. One of the reasons may be that AO research requires interdisciplinary knowledge and there exists a wide gap between the membrane field and biomedical technology. Membrane researchers are primarily from the nonbiomedical disciplines, and vice versa.

Although the artificial kidney technology can be considered as mature clinically, other types of membrane-based AOs, such as artificial lung, liver, and pancreas, still suffer from many technical challenges that must be resolved urgently. Needless to say, such challenges are very complex, requiring interdisciplinary collaboration across the fields of chemistry, biology, materials, and engineering. In addition, the quality of life of organ failure patients, albeit improved, is still poor. The AO technology must continuously improve not only from the membrane perspective, but also from the biocompatibility fronts to improve the quality of life and well-being of the patients.

Hence, in this review, in an attempt to narrow the gap between the membrane research and biomedical fields, the current status and challenges of membrane technology applied to artificial organs were compiled. More specifically, this review was written from a membrane researcher perspective, in order to aid and to facilitate future research on better membrane materials and module design.

## 2. Membrane-Based Artificial Organs

It is very important to clearly distinguish the key roles of an organ. Most organs are multifunctional and they perform physical, chemical and biological roles simultaneously with incredible precision. Generally, the physical roles of an organ can be partly or fully augmented with membrane technology; however, currently, the chemical and biological roles cannot be replaced in most cases. 

### 2.1. Artificial Lung (Blood Oxygenation Membranes)

**Figure 2:**
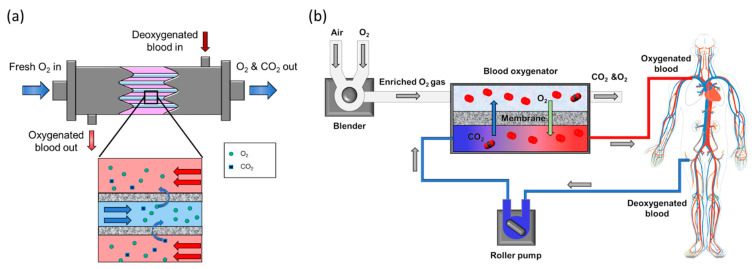
(**a**) Hollow fiber artificial lung schematic. The blood flows through the shell side of the hollow fiber module, and air (enriched oxygen) is supplied into the bore side. (**b**) Extracorporeal membrane oxygenation (ECMO) device schematic.

The lungs are part of the respiratory system, and their main function is to oxygenate the bloodstream while removing carbon dioxide in tandem. Hence, the lungs can roughly be considered as a gas exchange membrane, and the physical functions of the lungs can be partly augmented using membrane technology. Similar to the lung alveoli, a membrane can provide an interface between the air (oxygen) and the blood to facilitate the exchange of gases (Figure 2a). 

One of the iconic artificial lung devices was the bubble oxygenator proposed by De Wall and Lillehei in 1955 [12,13]. However, bubble oxygenators led to unwanted hemolysis (i.e., lysis or breakage of red blood cells) and air embolism, which was overcome with the use of membranes. Continuous improvements in the membrane permeance performance reduced the required surface area dramatically from 25 m^2^ of multilayer flat sheet membranes [14] down to about 2 m^2^ in the form of hollow fiber membranes [15,16]. Currently, the bottleneck is not caused by the membrane gas transport performance, but the blood side mass transfer resistance limits the blood oxygenation efficiency (discussed in detail below).

The artificial lung technology can be roughly split into two categories: a short-term and a long-term respiratory support. The short-term supports include open-heart bypass surgery, of which about 1.5 million cases are performed annually [9]. The venous blood from the patient is circulated extracorporeally (i.e., outside the body) using a heart–lung machine, also known as the cardiopulmonary bypass system (CPB). The blood gets oxygenated inside a membrane module (i.e., oxygenator) then returns to the patient. The operation lasts about six hours, and the purpose of the CPB is to temporarily augment both the heart and lung functions. 

The long-term respiratory support is carried out using the extracorporeal membrane oxygenation (ECMO) system. This treatment is reserved for severe lung failure patients, due to either aging, chronic lung diseases, or acute virus infection (e.g., MERS and COVID-19) [17], where mechanical ventilator treatment cannot supply the necessary oxygen demand. The components of an ECMO are similar to those of a CPB, but the term ECMO is generally reserved for long-term respiratory treatment (days to weeks). The main purpose of the ECMO system is to provide support until the body naturally recovers (bridge-to-recovery) [18]. Its clinical efficiency has been proved many times, as it can increase the survival rate of the patients by up to 75% relative to conventional treatment [17,19,20,21,22,23,24,25,26,27,28].

#### 2.1.1. Blood Oxygenation Physiology and the Role of Membrane

A set of adult human lungs is composed of hundreds of millions of alveoli that can exchange O_2_/CO_2_, with the effective surface area of approximately 70 m^2^ [29]. The O_2_/CO_2_ exchange rate of an average adult human lung is in the range of 200–250 mL/min (at rest) to 6 L/min (active work-out) [30]. 

**Figure 3:**
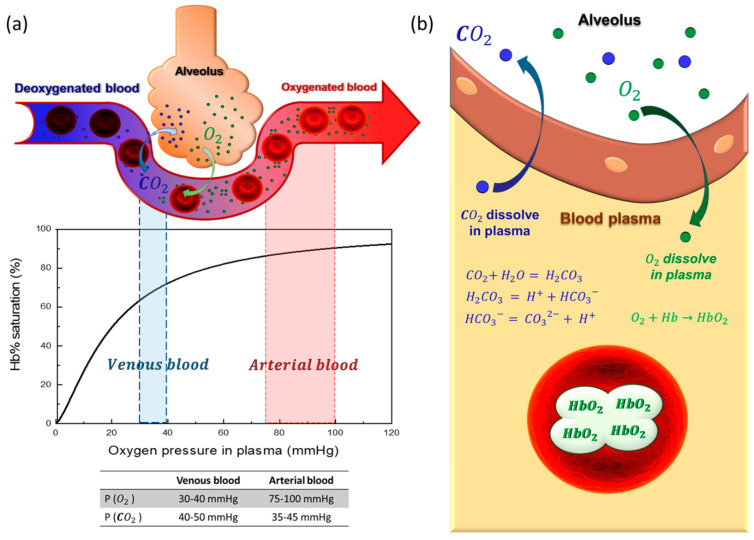
(**a**) The blood saturation curve predicted by the Hill model, approximate partial pressures of oxygen and carbon dioxide are given for venous and arterial bloodstreams. (**b**) The difference in gas exchange mechanism for oxygen and carbon dioxide inside an alveolus.

The exchange of gases between atmosphere and blood follows series of steps. The atmospheric oxygen is first inhaled into the lung, which then diffuses through the alveolus wall into the blood (Figure 3a). The oxygen then dissolves into the blood plasma, and is subsequently taken up by red blood cells. The dissolved oxygen inside red blood cells then binds to the hemoglobin proteins. The oxygenated blood is then carried around the body to aid cell aerobic metabolism. On the other hand, CO_2_ gas follows the opposite path (Figure 3b), except that most of the CO_2_ is carried by blood as carbonate and bicarbonate ions, not bound to the hemoglobin proteins (only about 5%).

The key difference between the two gases is the solubility (Figure 3b). The oxygen is only sparingly soluble in blood plasma (Henry’s constant of 0.0031 mL/mmHg O_2_/dL of blood), necessitating an oxygen carrier protein known as hemoglobin. The amount of dissolved O_2_ in plasma is commonly expressed in partial pressure unit. Assuming equilibrium, the partial pressure of oxygen can be conveniently correlated with the blood saturation level by the Hill equation [31,32]

(1) $$\mathrm{Hb}\;\mathrm{saturation}\;\left(\%\right)=100\frac{Kx^{\propto }}{1+Kx^{\propto }}\;$$

where x (mmHg) is the partial pressure of O_2;_ and K and ∝ are the parameter values (K=0.01455 and ∝ =1.405) [31].

**Figure 4:**
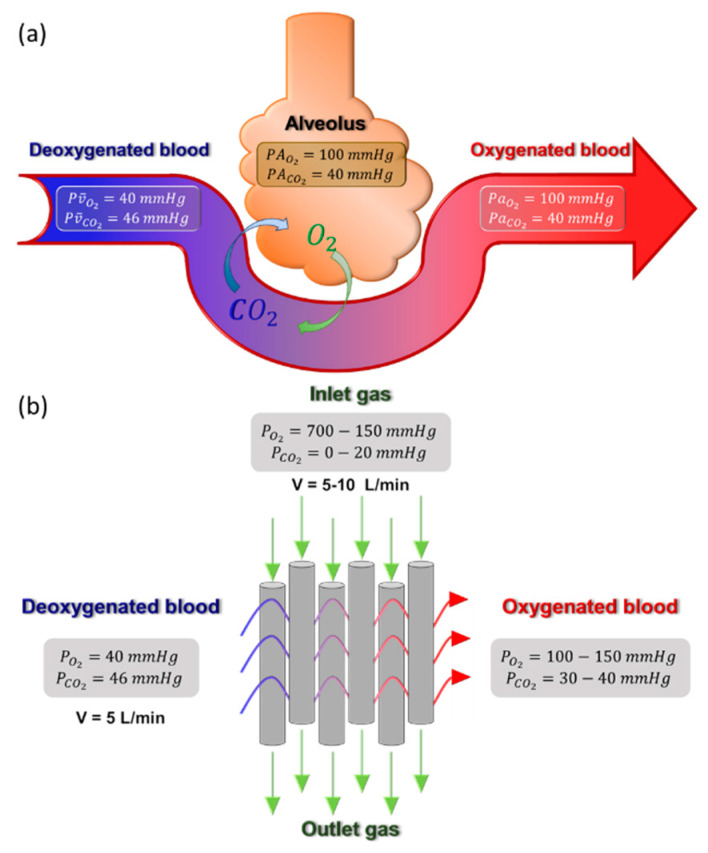
Illustration of gas transfer mechanism in (**a**) the human lung; and (**b**) membrane-based blood oxygenation device.

Hence, by measuring the oxygen concentration in blood plasma, the hemoglobin saturation percentage of the blood can be estimated [33]. The minimal metabolic O_2_ requirement of an adult is about 11 mmol/min, and approximately 9 mmol/min of CO_2_ must be removed from the body [34]. Hence, the CO_2_/O_2_ exchange ratio needs to be about 0.8, and the artificial lung performance must be carefully tuned to meet this exchange ratio to avoid unwanted hyper- or hypocapnia [34]. The maximum driving force attainable in normal physiological condition and in membrane module is graphically illustrated in Figure 4.

Compared to the alveoli conditions, the blood is exposed to steeper partial pressure gradients of O_2_ and CO_2_ in oxygenator membranes (Figure 4b). The deoxygenated blood enters the membrane with the partial pressures of O_2_ and CO_2_ at 40 and 46 mmHg, respectively. The feed gas flows through the bore side of the oxygenator membrane, and CO_2_/O_2_ exchange occurs at the membrane–blood interface. The oxygenated bloodstream exits the membrane with the partial pressures of 100–150 mmHg for O_2_ and 30–40 mmHg for CO_2_ [34]. It is important to note here that the driving force for CO_2_ exchange is lower than that of O_2_. Hence, the gas flowrate must be sufficiently high to meet the CO_2_ removal requirement and to satisfy the CO_2_/O_2_ exchange ratio of 0.8. It will be stressed several times in this review that the membrane permeance is not the bottleneck in the current artificial lung technology. Instead, fixing the exchange ratio to 0.8 is more of an engineering aspect of controlling the driving force for gas exchange. A comparison of the key parameters of human lungs and current oxygenator membranes is summarized in Table 1.

Similar to the lung physiology, the blood oxygenator system also consists of three phases: gas phase, membrane phase and blood phase. The most important parameter is the oxygen transfer rate, N (mol s^−1^) within the membrane oxygenator. The total oxygen transfer rate can be calculated by measuring the oxygen partial pressures of inlet and outlet bloodstreams (using a blood gas analyzer device). It can also be represented from the membrane perspective using the overall mass transfer coefficient, K (mol m^−2^ s^−1^ Pa^−1^), as follows:

(2) $$N=KA\;\Delta P_{LM}$$

where A (m^2^) is the effective membrane surface area, $\Delta P_{LM}$ (Pa) is the logarithmic mean difference of oxygen partial pressures. 

The oxygen transfer rate, N, is directly proportional to the mass transfer coefficient, and by definition, inversely proportional to the sum of resistances within the membrane oxygenator. The total mass transport resistance $R_{total}$ contains three main resistances: gas phase resistance, membrane phase resistance, and blood phase resistance.

(3) $$R_{total}=\;R_{G}+R_{M}+\;R_{B}$$

The total mass transport resistance $R_{total}$ can also be written as:

(4) $$\frac{1}{K}=\;\frac{1}{K_{G}}+\;\frac{1}{K_{M}}+\;\frac{1}{K_{B}}=\;\frac{1}{K_{G}}+\;\frac{\delta }{B}+\;\frac{1}{K_{B}}$$

where $=\frac{1}{K}$; G, M and B are gas, membrane and blood phases, respectively. δ (m) is the membrane thickness, and B (mol m m^−2^ s^−1^ Pa^−1^) is the permeability of the membrane.

**Figure 5:**
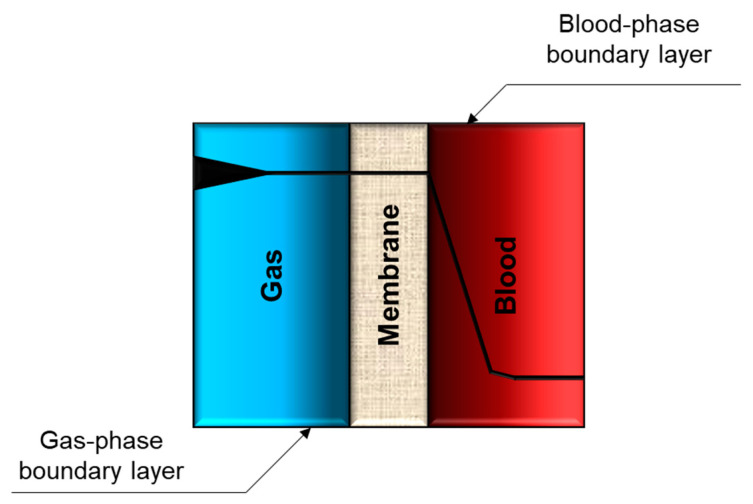
Series of resistances within the blood oxygenation device.

The gas transfer rate of the oxygenation device can be improved by reducing these three mass transport resistances (Figure 5). Among the resistances, the gas phase boundary layer resistance is negligible [9,34,36]. On the other hand, the blood phase boundary layer resistance can be as high as 100 times larger than the membrane resistance [9]. Hence, for current hollow fiber modules, the total mass transfer coefficient of the device K can simply be approximated to the mass transfer coefficient of the blood boundary layer $K_{B}$ [36].

**Figure 6:**
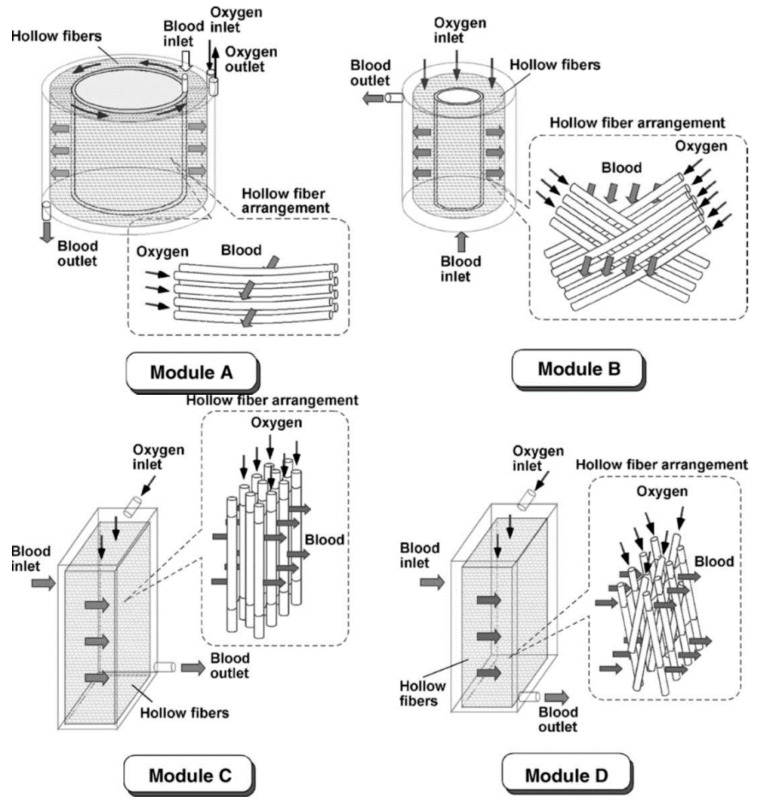
Hollow fiber arrangement in parallel (module) (**A**,**C**) and in perpendicular (module) (**B**,**D**); blood flow direction in coiled construction design (module) (**A**,**B**) and in rectangular solid design (module) (**C**,**D**). (Reprinted with permission from [36].).

The above analysis clearly shows that the focus must be given to lower the blood phase resistance, by providing better mixing for the bloodstream. Although higher velocity can give better mixing, it is also important to consider the pressure drop along the oxygenator, as high shear stress can cause blood trauma, which leads to unwanted hemolysis. There is a tradeoff between gas transfer rate and blood trauma. Many studies have also been carried out to optimize oxygenator geometries that give more complex flow patterns [36,37,38,39,40], as shown in Figure 6.

The mass transfer performance can be analyzed and cross-compared using the Sherwood number (*Sh*)—a dimensionless parameter as a function of the Reynold number (*Re*) and the Schmidt number (*Sc*). The Sherwood number for hollow fiber membrane module can be expressed as follows [36]:

(5) $$Sh=f\;\left(Re,\;Sc\right)=a\;Re^{b}\;Sc^{c}$$

The Sherwood number can be also written as:

(6) $$\frac{k_{B,L}\;d_{0}}{D_{B}}=a\;{\left(\frac{d_{0}\;u_{0}}{v}\right)}^{b}{\left(\frac{v}{D_{B}}\right)}^{c}$$

where $k_{B,L}$ (m s^−1^) is the mass transfer coefficient which depends on the oxygen concentration in liquid ($k_{B,L}=Hk_{B}$); $d_{0}$ (m) is the outer diameter of hollow fiber membrane, $u_{0}$ (m s^−1^) is the superficial velocity of blood, v (m^2^ s^−1^) is the kinematic viscosity, and $D_{B}$ (m^2^ s^−1^) is the diffusion coefficient of oxygen in blood. 

Moreover, the Sherwood number for blood flowing in a thin channel, such as a microfluidic device, can be expressed as follows:

(7) $$Sh=a\;Gz^{b\;}=\left(\frac{d_{h}}{L}\right)\;Re\;Sc$$

where *Gz* is the Graetz number, a dimensionless number represents for the laminar flow in a conduit, $d_{h}$ (m) is the hydraulic diameter, and L (m) is the length of fiber. The Sherwood numberdepends on the number of hollow fiber in a bundle or the fiber orientation in the device (Table 2). 

Many experiments on various configurations have been conducted to obtain reliable Sherwood correlations. Generally, the gas transfer rate is better for the transverse flow compared to the parallel flow configuration. More recently, a generalized model for both the transverse and parallel flow for the oxygenators was reported to predict the oxygenation performance [37], as summarized in Table 3. The authors showed that the oxygenator void fraction of hollow fiber bundles ($\varepsilon_{f}$) affects the mass transfer coefficient in several ways. For example, when $\varepsilon_{f}$ is between 0.3 to 0.5, the mass transfer coefficient of the square fiber arrangement with perpendicular flow is better than the staggered arrangement. However, for the parallel flow, the behavior tends to be the opposite [37].

#### 2.1.2. Membranes for Artificial Lung

Currently, the two main polymers used for artificial lung membranes are polypropylene (PP) and poly-4-methylpentene (PMP). The first generation artificial lung is a PP-based microporous membrane with hydrophobic characteristics. These membranes are highly porous with excellent gas permeance but have a short lifetime of about six hours [41,42], after which the membrane gets wet. They are mostly fabricated via the melt-extrusion-stretching method, which gives an average pore size between 0.05 to 0.1 µm. Another widely employed fabrication method is the TIPS process (i.e., thermally induced phase separation, which gives narrower pore size distribution [43].

As the PP membrane is intrinsically hydrophobic, the blood only contacts the surface of the membrane to facilitate the gas exchange; hence, the blood does not (and should not) leak through the membrane [41]. This type of membrane process is also commonly known as the membrane contactor process. For the open-heart surgery application, the microporous membranes are reliable. However, in long-term treatment, the plasma proteins adsorb onto the membrane surface, which increases the surface energy of the membrane material, eventually allowing unwanted membrane wetting and allowing the blood plasma to break through [41,44,45].

In the early 2000s, the second generation artificial lung membrane was developed based on the PMP material with a remarkably longer lifetime (last 2-4 weeks) [46,47,48]. Compared to the porous PP membrane, PMP membranes have a thin dense layer that can effectively prevent the plasma leakage [46,48]. As PMP is generally insoluble in organic solvents, these membranes are also prepared via melt-extrusion or TIPS method. From the membrane technology perspective, the formation of such skin layer with TIPS is interesting, as the thin skin layer of a semicrystalline polymer can only be formed via combination of NIPS (nonsolvent-induced phase separation) and TIPS technique, so called N-TIPS method [43,49]. 

Expectedly, the presence of a thin dense layer significantly reduces the gas permeance. Compared to the porous PP membrane, where gas molecules can freely permeate through, PMP membranes with a dense skin layer exhibit two orders of magnitude lower gas permeance as the gas molecules must diffuse through the dense PMP material. [45]. Nevertheless, compared to other polymeric materials, PMP has high intrinsic gas permeability, as summarized in Table 4. It is important to clearly distinguish the term membrane gas permeance from material intrinsic gas permeability. The gas permeance is typically in the unit of GPU (gas permeation unit) and it represents the performance of a membrane. On the other hand, the gas permeability is in the unit of barrer, and it characterizes the intrinsic gas permeability of a dense material. 

Additionally, as discussed previously, the membrane permeance is not the bottleneck in the process of blood oxygenation. Hence, even with such low permeance, the blood oxygenation efficiency is not compromised. PMP also exhibit good hydrophobicity, preventing the unwanted wetting of the membrane by the blood plasma. Due to these advantages, PMP membranes can be applied to long-term treatment including ECMO, continuously for more than 42 days [39,40].

More recently, third generation artificial lung membranes have been proposed using fluoropolymers [50]. The characteristic low surface energy of fluoropolymers prevents unwanted protein adsorption onto the membrane surface, potentially improving the long-term hemocompatibility of artificial lung membranes. Among the known fluoropolymers, polytetrafluoroethylene (PTFE), also known by its tradename Teflon, exhibits outstanding characteristics such as low surface energy, chemical stability, mechanical rigidity, and high biocompatibility. However, it is technically difficult to process PTFE polymer into a thin, hollow fiber shape due to its high melt viscosity. 

Kim et al. [50] have shown that other fluoropolymers with better processability, such as PVDF and P(VDF-co-HFP), can be processed into membranes with target pore size. Apart from these options, many possible possibilities exist in the realm of fluoropolymers, yet to be explored for artificial lung membranes. 

#### 2.1.3. Current Challenges and Research Direction

It has been clinically proven that the artificial lung treatment can increase the survival rate of lung failure patients [54]. Despite such improvements, many challenges remain to further improve the survival rate and quality of life of the patients. Future membrane research must focus on three fronts: (1) better hemocompatibility, (2) better module design with lower prime volume, and (3) smaller and more portable systems. 

##### Blood Compatibility (Hemocompatibility)

The concept of blood compatibility, or hemocompatibility, has been one of the most daunting challenges in the field of biomaterial research. In spite of 50 years of intensive research, biomaterial researchers even coined the term “blood compatibility catastrophe” [55] to show that there is no consensus as to which materials are even “blood compatible.” The term biocompatibility is a broader concept that includes hemocompatibility, and these two terms are often used interchangeably in the blood-contacting materials literature. 

**Figure 7:**
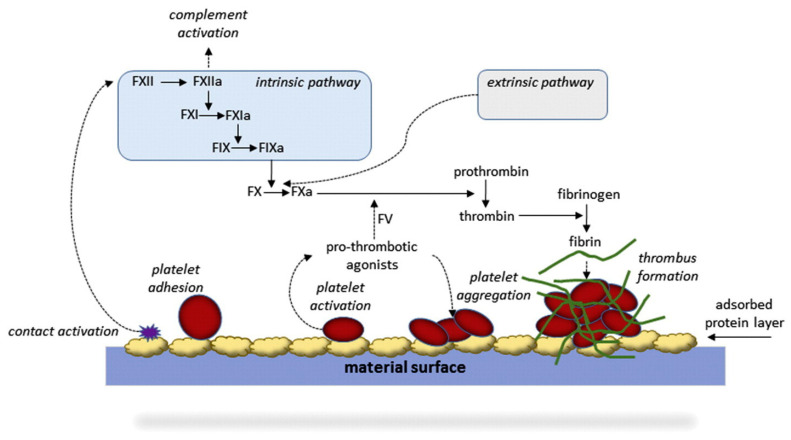
Simplified coagulation response by the contact activation of blood proteins. (Reprinted with permission from [62]).

Inside an artificial lung membrane module, the blood comes in contact with the membrane (among other parts), and the proteins inside the blood, such as albumin, fibrinogen, fibronectin, and many others, immediately bind to the surface. As illustrated in Figure 7, the initial adsorption step sets off cascades of complex immune response and blood clot formation (e.g., thrombosis) processes [18,56,57,58]. Hence, the protein–material interaction is an important indicator of hemocompatibility.

The key issues in artificial lung membranes are the formation of blood clots and inflammatory response. These mechanisms, although vital for our survival, can cause a variety of adverse issues during clinical treatments. Particularly, blood clots can block the blood pathway and dramatically lower the oxygenation efficiency. Current state-of-the-art artificial lung membranes can last for a few weeks before blood clots (thrombosis) adversely affect patients [59,60,61].

As the protein adsorption is the first step that initiates both the blood coagulation pathway and the inflammatory response [63], many works have focused on minimizing the protein adsorption onto blood-contacting devices [50,64,65,66,67]. Generally, proteins bind to a surface via hydrophobic interaction, and hence the hydrophilic modification of a surface effectively lowers the protein adsorption, which in turn improves the hemocompatibility [62]. However, artificial lung membranes require hydrophobic properties to avoid pore wetting and plasma breakthrough. This is precisely the reason why the current artificial lung membranes are mostly based on hydrophobic materials such as PP and PMP. 

**Figure 8:**
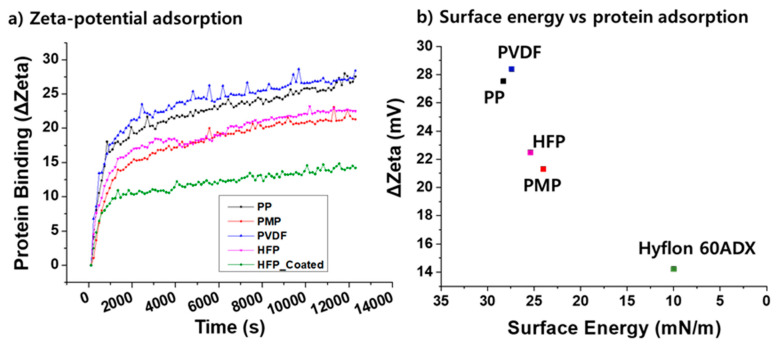
(**a**) Rate of protein adsorption measured by streaming zeta-potential device, (**b**) inverse correlation between material surface energy and the amount of protein adsorption. (Reprinted with permission from [50].).

Another strategy to prevent the protein adsorption, particularly for artificial lung membranes, is to minimize the surface energy. It has been shown that the amount of protein adsorption is inversely proportional to the membrane surface energy [50]. Figure 8 clearly shows that a fluorinated surface, although hydrophobic, can effectively prevent protein adsorption and potentially improve the hemocompatibility. Hence, fluorinated polymers could be a promising alternative to the current PP and PMP materials.

**Figure 9:**
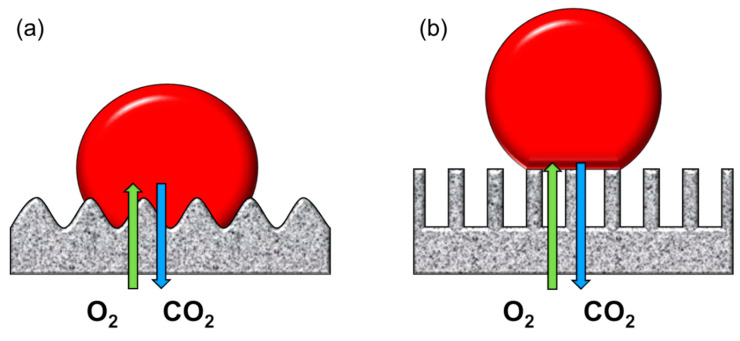
The wettability of blood droplet on the rough surface: (a) Wenzel state, (b) Cassie–Baxter state.

Protein adsorption can be further inhibited with super liquid-repellent surfaces, also known as superamphiphobic surfaces [50,67,68,69,70]. The superarmphiphobic surface can only be realized with appropriate surface roughness modification and surface energy modification. The first step is to implement the right roughness with nanostructures in order to create the capillary force at the blood–surface interface [71,72,73]. The nanostructure creates an air gap between the liquid and the surface of the material, transforming the surface from the Wenzel state to the Cassie–Baxter state [71,74,75,76,77,78,79,80] (Figure 9). There are many ways to fabricate nanostructures, such as spray particle coating [57,81,82,83] or etching methods (plasma, laser, chemical, and electrochemical etching) [71,78,84,85,86,87,88,89,90,91].

To realize a superamphiphobic surface, the surface energy must also be minimized in tandem, typically using fluorochemicals. The most commonly used ones are fluorocarbon-based compounds, (-CF_2_-)_n_, where *n* is between 4 and 10. These perfluorochemicals exhibit extremely low surface energy but can be toxic to the environment and health [92], and cannot be applied for healthcare materials. Alternative perfluorochemicals are being sought which are safer but with similar low surface energy characteristics [92]. The lowest surface energy reported is 9.3 mN/m using fluorinated polyhedral oligomeric silsesquioxanes (POSS) [93]. 

Apart from controlling the protein adsorption, hemocompatibility can be achieved by controlling other factors within the blood physiology. For example, blood coagulation cascades can be inhibited using nitric oxide (NO) [94] or by coating heparin derivatives [95]. Coating the membrane with phosphorylcholine (PC) compound has also been reported to decrease thrombosis [96,97,98]. A considerable reduction of platelet adhesion was reported when coating the membrane with poly(2-methoxyethylacrylate) (PMEA) [99,100]. One of the most promising methods is to form a thin endothelial cell layer on the membrane surface to mimic the actual blood vessels. Hypothetically, a monolayer of endothelial cells on membranes can achieve complete hemocompatibility [101]. Many interesting pieces of research have been carried out [101,102,103,104,105,106,107], but its efficacy and clinical safety must be proven before it can be implemented in commercial products.

Despite several decades of hemocompatible research, it is as yet difficult to obtain membranes that can completely avoid blood coagulation and inflammation. In fact, this challenge is not only in the artificial lung field, but in the entire discipline of blood-contacting biomaterials. Many promising developments are being reported but have yet to be implemented into artificial lung technology. 

##### Improving Blood Oxygenation Efficiency

The performance of current commercial ECMO modules is summarized in Table 5. It can be seen that there is still a wide gap between the oxygenator membrane performance and that of human lungs (Table 1). There are several ways to improve the oxygenator membrane performance. First, the blood oxygenation efficiency can be improved by optimizing the blood’s fluid dynamics within the module. Many articles reported different strategies for better module designs [39,108,109,110,111,112]. Most of the blood oxygenator modules are designed with hollow fiber bundles with the blood flowing outside the fiber membranes. In this way, the contact area and the gas transfer efficiency of the artificial lung can be maximized [39,109]. In order to avoid unwanted blood trauma due to shear flow [113], computational fluid dynamics (CFD) tools have been applied to optimize the module design and to improve the ECMO performance [110,112,114,115]. 

Secondly, the prime volume, or the amount of extracorporeal blood, must be minimized. Simultaneously, the contact area and contact time with foreign materials must also be minimized to improve the blood hemostasis. One obvious strategy is to minimize the hollow fiber thickness (100 μm) similar to that of alveolus thickness (2 μm), but materials with strong enough mechanical properties must be developed. Theoretically, this strategy could lower the prime volume quite significantly.

**Figure 10:**
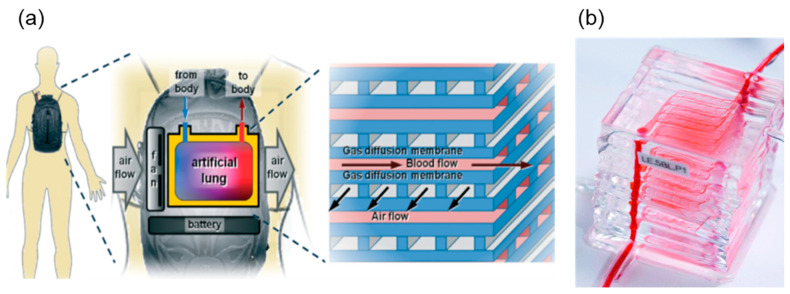
(**a**) A conceptual drawing of an initial clinical application of microfluidic artificial lung technology—an ambulatory pumpless extracorporeal lung assist system (reprinted with permission from [128]); (**b**) a multilayer PDMS microfluidic blood oxygenator (reprinted with permission from [131].).

Apart from the hollow fiber membrane modules, other types of artificial lung are being developed, such as paracorporeal, introthoracic and intravenous artificial lungs [116,117,118,119], some reaching clinical trials [120]. In particular, microfluidic devices are now considered as the most promising artificial lung platform for the future [121,122,123,124,125,126,127]. Mostly fabricated with PDMS material, the membrane thickness can be controlled within 6 to 130 μm [128], and the oxygen exchange rate can reach as high as 329 mL·min^−1^·m^−2^ [129] (Figure 10). In addition, the blood paths can be customized to maximize the contact area and residence time.

However, main challenges of the microfluidic artificial lung are, again, related to hemocompatibility [128]. Additionally, although the photolithographic fabrication step can be cumbersome, the recent development of the PDMS fabrication technique could lead to a breakthrough [130].

##### Wearable and Implantable Artificial Lung

To improve the quality of life of lung-failure patients, the device must become smaller and more portable. For instance, a wearable or implantable artificial lung could be a preferred treatment. Several different types of such devices have been reported. In 2012, researchers reported results on a wearable artificial lung, tested with sheep for 30 days with stable oxygen saturation levels [132]. In 2019, a paracorporeal ambulatory assist lung (PAAL) was tested with sheep for six hours [133] and for five days [134]. Another wearable ECMO device, compliant thoracic artificial lung (cTAL), was tested on sheep for 14 days [135]. Additionally, intravenacaval oxygenator and carbon dioxide removal device (IVOX) [136] and the Hattler respiratory gas exchange catheter [137] were also reported but these artificial lungs failed during clinical tests. The wearable and implantable artificial lung devices are still in the early stages of development. Expectedly, the main challenge is the hemocompatibility of the materials. In order to achieve safety over the long-term, better understanding of the blood–material interaction is required.

### 2.2. Artificial Kidney and Blood Purification

The kidneys are bean-shaped organs in the renal system, located on both sides of the lower spine. They are made up of nephrons that act as a physical filter to remove biological waste and excess water from the blood to maintain homeostasis. In addition, human kidneys perform biological roles, such as hormone production, to make new red blood cells (erythropoietin), to promote bone health, and to regulate blood pressure. These biological roles cannot be augmented with membrane technology yet.

**Figure 11:**
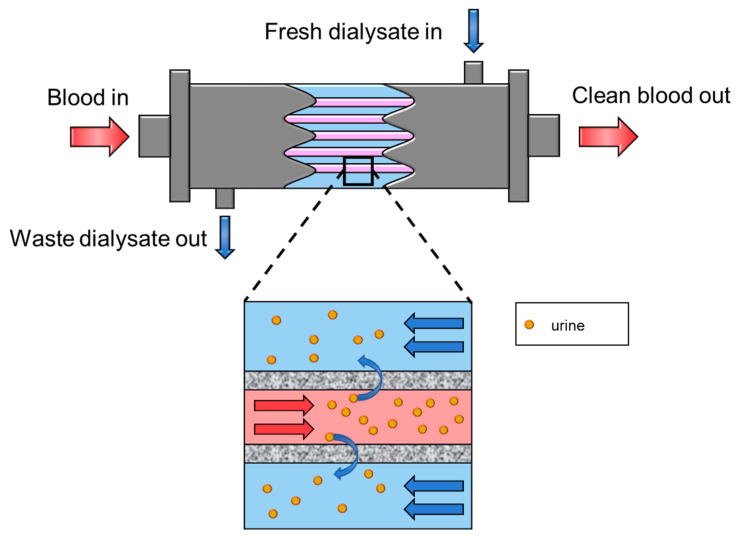
Membrane-based artificial kidney schematic.

In the case of kidney failure, the patient must be treated with artificial kidney technology at least twice a week for blood purification. The artificial kidney, also known as the dialyzer (Figure 11), is one of the most important technologies that helps millions of people around the world. The term “dialyzer” was first coined in the middle of the 19th century [138], but the first real success in prolonging a patient’s life was reported in 1945 [139]. Since then, artificial kidney membranes underwent tremendous improvements in treating renal diseases (i.e., kidney failures). After many module designs, such as coil and plate types, the current state-of-the-art artificial kidney membranes are mostly in the form of hollow fiber modules.

**Figure 12:**
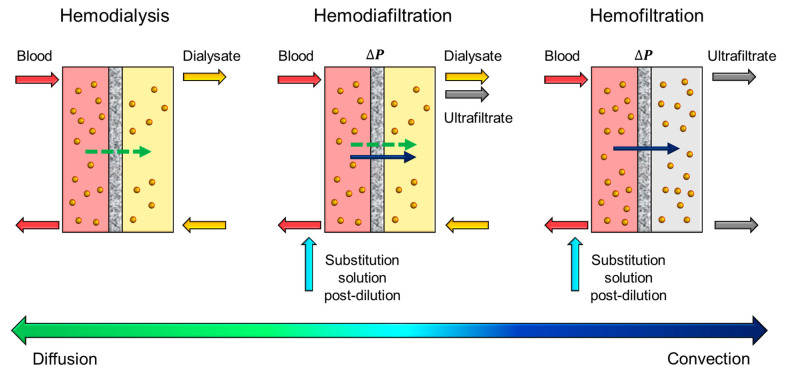
Schematic illustration of hemodialysis, hemodiafiltration and hemofiltration.

One of the key roles of artificial kidney is to purify blood, also known as extracorporeal (e.g., outside the body) blood purification, and it can be further classified as hemodialysis (HD), hemofiltration (HF), and hemodiafiltration (HDF). As illustrated in Figure 12, hemodialysis is a blood purification method that requires a separate dialysate stream, and toxins are extracted from the blood into the dialysate stream via natural diffusion down the concentration gradient. Normally, the MWCO (molecular weight cut off) of dialyzer membranes is from 3000 Da to more than 15 kDa [140,141]. In recent times, the super high flux dialyzer membrane can have a MWCO of around 65 kDa [142]. It is interesting to note that dialysate production occupies a considerable market portion of the reverse osmosis membranes, as ultrapure water is required to make the dialysate solution. 

Hemofiltration is similar to hemodialysis, but a transmembrane pressure is applied across the membrane to push the waste solutes and water through the membrane. Unlike hemodialysis, hemofiltration does not require a dialysate stream, but a substitution fluid is used to make up the lost blood volume. Additionally, as the applied pressure induces liquid convection through the membrane, hemofiltration can permeate large solutes faster than hemodialysis [143]. On the other hand, hemodiafiltration is a hybrid of the hemodialysis and hemofiltration methods. This method can improve the permeance by applying the pressure gradient and increase the performance of the membrane [144].

Currently, there are many different types of polymers to fabricate dialysis membranes, and it can be divided into five different groups—namely, cellulose derivatives, polysulfone derivatives (PSU), polyacrylontrile (PAN), polymethylmethacrylate (PMMA) and ethyl-vinyl-acetate copolymer (EVAL). 

The first group is the cellulose derivatives. Most of the hemodialysis membranes were made from cellulose materials in the early days (around 1950) of artificial kidney technology. In 1985, Henderson et al. [145] published an article which reported concerns over the blood biocompatibility of the regenerated cellulose membranes used for artificial kidneys [145]. This paper initiated researchers searching for new biomaterials with higher biocompatibility to apply in dialysis membranes. Nowadays, synthetic membranes with better biocompatibility have mostly replaced the cellulosic membranes. As of 2016, synthetic membranes such as polysulfone (PSU), polyethersulfone (PES), PAN, PMMA and EVAL make up 95% of the dialysis membrane market share [9]. Interestingly, acetylated cellulose polymers, such as cellulose triacetate (CTA), exhibit reasonable biocompatibility [146] and hence still hold 5% of the dialysis membrane market [147]. 

The second group is the polysulfone-based polymers such as polysulfone (PSU) and polyethersulfone (PES), and they currently dominate the hemodialysis market. PSU-based dialysis membranes are proven to have higher biocompatibility and better clinical performance. Long years of research have shown that a delicate balance is required between hydrophilic and hydrophobic properties to obtain adequate biocompatibility. As PSU polymers are intrinsically hydrophobic, researchers have blended polyvinylpyrrolidone (PVP) additives to increase the PSU membrane hydrophilicity, and its positive effect on biocompatibility have been clinically proven [148].

The third group, PAN dialysis membranes were first reported in 1972 by the Rhone-Poulenc company as “AN69” with high flux. The AN69 was made with PAN and hydrophilic sodium-meth-allyl-sulfonate additives with high biocompatibility [149]. The fourth group is PMMA hemodialysis, which is known as a biocompatible adsorbent membrane [150]. PMMA membranes show excellent adsorption of biological middle molecule (β_2_ microglobulin) at the wall and the inner surface of membranes [150]. Lastly, EVAL and EVOH polymers are promising dialysis materials with remarkable blood compatibility, based on their strong hydrophilic properties. Notably, protein adsorption onto the EVAL membrane is extremely slow [151].

**Figure 13:**
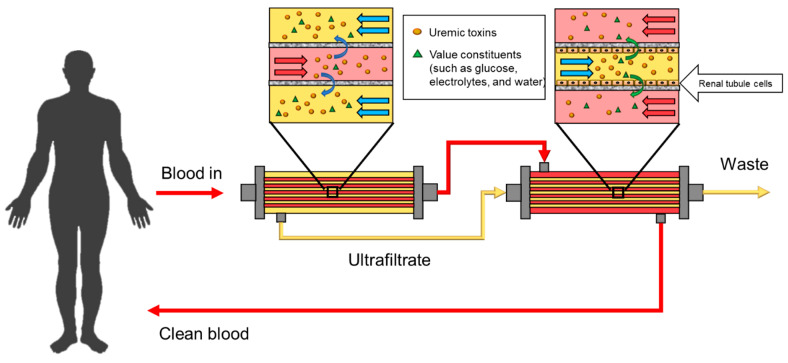
Bioartificial kidney (BAK) schematic.

The cell-free artificial kidney system has been employed for a long time, and the basic principle of the dialysis system is to eliminate the toxins from the blood using a membrane. However, this system cannot replace the endocrine, homeostatic, metabolic and regulatory functions of the human kidney (biological and chemical functions). Hence, researchers have tried to combine the polymeric membranes with kidney cells, known as the bioartificial kidney (BAK). The first BAK prototype was presented in 1987 [152]. A BAK device is composed of two parts (Figure 13): a hemofiltration device and a renal tubule assist device (RAD). A patient’s blood is first separated by a hemofilter into the blood and the ultrafiltrate streams. Then, both streams enter the RAD, where the valuable components in the ultrafiltrate are reabsorbed into the bloodstream. A hollow fiber module incorporating renal cells is used. The commonly employed polymers are PSU [153,154], PAES [154] and PES [155]. 

The source of renal cells can be from porcine [156,157], canine [158] or human renal cells [153,154,155]. More recently, 3D printing technologies were applied to mimic a more kidney-like structure [159] in combination with the microfluidic technology [160]. On the other hand, wearable artificial kidney (WAK) devices are actively being developed to improve the quality of life of the patients while reducing the financial burden of long-term treatments [161,162,163]. An ideal WAK devices should weigh under 8 kg and the total volume should be less than 0.1m^3^ [164]. Several urea removal strategies have been reported in the WAK such as sorbents [165], photo-oxidation [166] and electro-oxidation [167]. An exciting work that applies artificial intelligence (AI) techniques to improve dialysis therapy has been reported by real-time feedback responses and life and death making decisions [168,169]. 

### 2.3. Artificial Liver

The liver is a multifunctional organ with more than 200 functions that is essential for survival. It is the largest internal human organ, weighting about 1.5 kg, and is composed mostly of hepatocytes (liver cells). The liver performs many essential metabolic functions, along with nutrient storage, blood detoxification, plasma protein synthesis, and hormone control [170]. 

Liver failure can be acute and/or chronic, and it can occur due to many reasons; common causes are the ingestion of a toxic substance, medicine, drug overdose, alcohol consumption, and viral hepatitis. Liver damage can lead to a variety of life-threatening physiologic and metabolic abnormalities like hemorrhage, hypoglycemia and hyperammonemia [171]. Fortunately, the liver is one of the unique organs that can regenerate, and it can recover itself from minor damages (e.g., binge drinking). However, as the roles of liver are primarily biochemical functions requiring hepatocytes, the most effective treatment for severe liver failure patients is, again, organ transplantation. 

Researchers have tried to develop various artificial liver support devices for liver failure patients. The current liver support systems can be divided into two main types: (1) nonbiological artificial liver systems that augment the blood detoxification role of the liver, and (2) bioartificial liver with hepatocytes that can also augment the biological roles.

The first type is the conventional artificial liver support which is a nonbiological membrane system—that is, without any cellular (hepatocyte) functions. Its main role is to augment liver detoxification function. To achieve blood homeostasis, two different classes of blood toxins must be removed from the body. There are low MW hydrosoluble toxins such as urea and ammonia. These toxins can be effectively removed by the kidneys or by hemodialysis. On the other hand, there are protein-bound toxins that are too big to be removed by kidney or hemodialysis. These protein-bound toxins must be treated by the liver prior to excretion. 

Some of the biological waste toxins (e.g., bilirubin, aromatic amino acids, endotoxins, etc.) are hydrophobic and sparingly soluble in blood plasma. These toxins in the blood are transported to the liver as protein-bound, mostly bound to albumin proteins. It is important to note here that albumin is the most abundant protein in the blood plasma with many important roles including osmotic pressure control and toxin scavenging.

Particularly, bilirubin is a chemical compound produced during the breakdown of old red blood cells. The produced bilirubin binds to albumin in the blood, and is transported to the liver, where it becomes conjugated with glucuronic acid, after which it can be degraded and excreted. The blood bilirubin concentration is commonly considered as an important marker for liver malfunction, as high bilirubin concentration can lead to hepatic encephalopathy (brain damage), or coma. Hence, the main objective of the current artificial liver systems is to eliminate these albumin-bound toxins in the blood, as this is the most urgent issue in the event of liver failure.

From membrane engineering perspective, the challenge is the difficulty in discriminating the albumin-bound toxins from other important chemicals inside the blood. These toxins (e.g., bilirubin and tryptophan) are strongly bound to albumin proteins, and albumin itself should not be removed from the bloodstream for homeostasis. The MW of albumin is approximately 69 kDa. It is possible to simply replace the entire plasma (plasmapheresis), but it is not practical as it requires large amounts of fresh plasma continuously [172]. Additionally, the blood can be pushed through an absorbent column (hemoperfusion) to remove toxins but this is relatively nonspecific (low selectivity) and it can inadvertently remove other important biomolecules in tandem [173]. 

**Figure 14:**
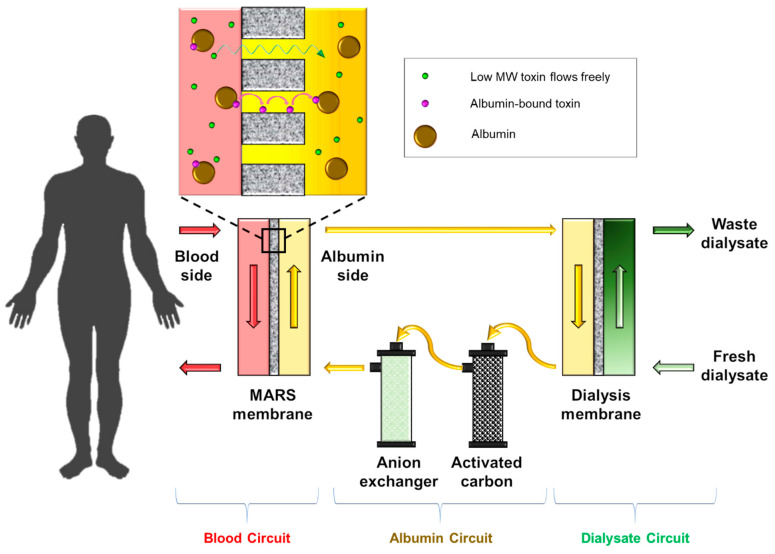
Simplified schematic of Molecular Adsorption Recirculating System (MARS).

Currently, two different strategies have been developed (Figure 14). The first strategy, known as MARS (molecular absorbent recirculation system), was first applied in 1996 and it utilizes a technique called albumin dialysis [174]. Although bilirubin exists as albumin-bound form in the blood plasma, it is strictly in thermodynamic equilibrium between the free bilirubin form and albumin-bound bilirubin form. Hence, the only way to selectively push free bilirubin through a membrane is by circulating highly concentrated free albumin (unbound) solution on the dialysate side of the membrane, as illustrated in Figure 14. Generally, 20 wt% albumin (unbound) solution is used as the dialysate. Hence, due to the free albumin concentration gradient, bilirubin unbinds itself from blood plasma albumin, diffuses through the membrane, then subsequently binds to a free albumin in the dialysate solution. For this process, a conventional hemodialysis membrane (albumin-impregnated) is used with MWCO of 50~60 kDa. Hence, this membrane blocks albumin permeation while allowing bilirubin (MW of 584 Da) to permeate freely. 

The driving force for bilirubin permeation is purely by the concentration gradient of the free albumin across the membrane. Hence, the phase equilibria of the albumin–toxin systems must be understood. It has been reported that the binding constants (K_eq_) for bilirubin and tryptophan, two of the most problematic liver failure toxins, are 10^7^ L/mole [175] and 10^4^ L/mole [176], respectively. These values indicate that basically all bilirubin and tryptophan molecules exist as albumin-bound form. Therefore, the toxin removal efficiency is primarily a function of albumin dialysate concentration. 

The albumin dialysate solution circulates around a loop and albumin is regenerated using two adsorption columns, mostly with an activated carbon column to adsorb uncharged toxins (e.g., tryptophan), and an anion exchange column to remove negatively charged compounds (e.g., bilirubin). Apart from bilirubin, other low MW compounds also permeate the MARS membrane into the albumin dialysate solution. Such compounds are then removed in the second dialysis membrane (Figure 14). Hence, MARS also performs artificial kidney functions, but not as effectively. This system can only last about 6–8 hours, after which the adsorption columns must be replaced. 

Another strategy of artificial liver system is called Prometheus, or FPSA (Fractionated Plasma Separation and Adsorption), first proposed in 1999 [177] and clinically commercialized in 2003 [174]. The key difference of this process compared to MARS is the use of a loose polysulfone membrane with MWCO of about 250 kDa (MARS employs a 65 kDa membrane). Instead of using albumin dialysate, the membrane allows the toxin-bound albumin proteins in the blood to selectively permeate. The albumin filtrate is then treated with adsorption resins to regenerate the albumin (toxin removed). The regenerated albumin stream is then recombined with the blood, and the bloodstream is further dialyzed with a conventional hemodialyzer. 

The clinical data for the MARS and Prometheus systems showed that the treatments clearly improve the biochemical homeostasis (e.g., bilirubin concentration), but unfortunately these treatments did not improve the survival rate of the patients [178,179]. Such an outcome may be because the liver performs many other important functions besides maintaining blood homeostasis. Hence, simply augmenting the blood detoxification role of the liver cannot guarantee the patient’s survival. Liver failure almost always leads to multiorgan failures which are, currently, difficult to predict and to treat. From membrane performance perspectives, the use of facilitated transport membranes can be applied to the MARS system to enhance the toxin removal rate, and membranes with better selectivity between albumin and fibrinogen could improve the Prometheus performance. 

There are also other artificial liver systems such as SPAD [180], and SEPET [9]; however, conventional artificial liver systems have clear limitations and their role is primarily to temporarily treat acute liver failure patients. These treatments cannot be applied in the long-term for severe liver failure patients. Hence, future research must focus on bioartificial liver (BAL) systems with hepatocytes that can also perform the biological roles of the liver, such as regulation and synthesis. 

**Figure 15:**
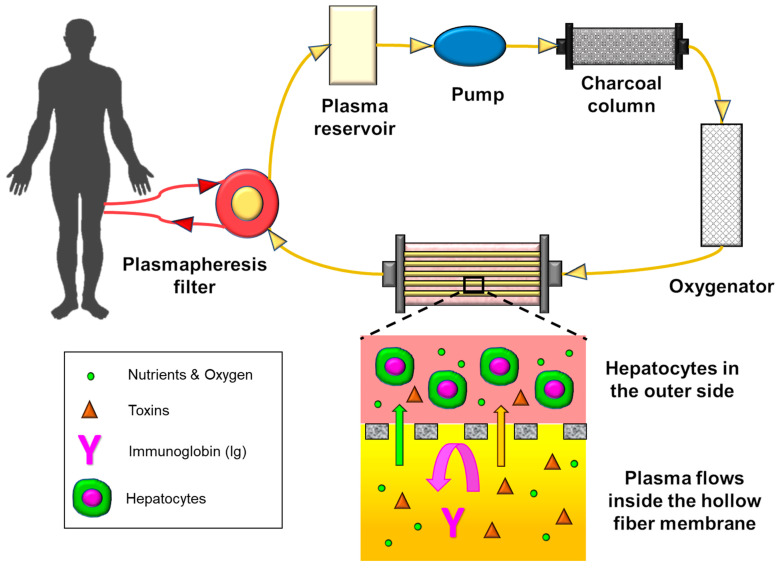
Bioartificial liver schematic.

A BAL system combines the membrane technology with a compartmentalized bioreactor that contains hepatocytes (Figure 15). The key role of the membrane in BAL is to protect the hepatocytes (foreign source) from the immune system. The membrane needs to prevent blood immune cells from passing through the membrane, while allowing other biomolecules and nutrients to permeate and come into contact with the hepatocytes; however, it should be noted that the membrane itself can also initiate the immune response and blood coagulation [181,182], and necessary measures (anticoagulation) must be implemented. Again, hemocompatibility is the key challenge.

Many BAL systems have been tested, such as ELAD (Extracorporeal Liver Assist Devices) [183], BLSS (bioartificial liver support system) [184], and BIOLIV A3A [185]. Although considerable improvements have been made, BAL systems are still in early stages of development with countless challenges. The development of BAL is very challenging as many parameters must be considered simultaneously, such as hepatocyte source, bioreactor design, immune response, and blood homeostasis. In addition, many difficult challenges must be overcome from the bioreactor component as well. 

From the membrane perspective, the rate of mass transfer of biomolecules through the membrane must be controlled to maintain hepatocyte survival and cellular metabolism, while effectively suppressing the immune response and blood coagulation. Hence, membrane properties such as the pore size, surface charge, and wettability must be optimized for this purpose [186,187]. The current BAL membranes are fabricated from rather conventional materials such as polysulfone [188,189,190,191], cellulose [192], and cellulose acetate [193,194]. Ideally, more application-specific materials must be developed. 

### 2.4. Bioartificial Pancreas

The primary role of the pancreas is to maintain glucose homeostasis in the blood by controlling the concentration of endocrine hormones (e.g., insulin and glucagon). Pancreas failure can lead to diabetes, one of the most well-known diseases in the world. Upon pancreas failure, the body loses control of its insulin production, causing various types of diabetes. Type 1 diabetes, which mostly occurs in young children, is caused by a pancreas disorder where insulin production cannot meet the body’s demand. On the other hand, type 2 diabetes, which is much more prevalent in adults, occurs when the body develops insulin resistance and the blood sugar level remains high for prolonged periods. 

There are several treatment methods to control the sugar levels and insulin concentrations for patients with the type 1 diabetes. The method can range from simple injection of the required insulin to cutting-edge islet (pancreatic cell) transplantation technology. Instead of the transplantation of the whole pancreas, only the islet (pancreas) cells can be transplanted from the donor, known as the Clinical Islet Transplantation (CIT) [195]. The CIT technology has been improving, with its five-year success rate increasing from only 15% to approximately 60% [196,197,198,199]. 

The bioartificial pancreas (BAP) technology, similarly to BAL, combines membrane technology with islet cell transplantation technology. A BAP consists of islet cells encapsulated within a semipermeable membrane capsule that acts as an immune system barrier [200,201]. 

An ideal BAP needs to meet the following requirements: (1) segregate and protect the islet cells from the immune system; (2) allow glucose, nutrients and oxygen to permeate, so that the response time to blood glucose level is minimized; (3) exhibit high hemocompatibility with minimal inflammatory response by the host body to ensure prolonged use inside the body; (4) easily implantable and retrievable in case of failures. 

**Figure 16:**
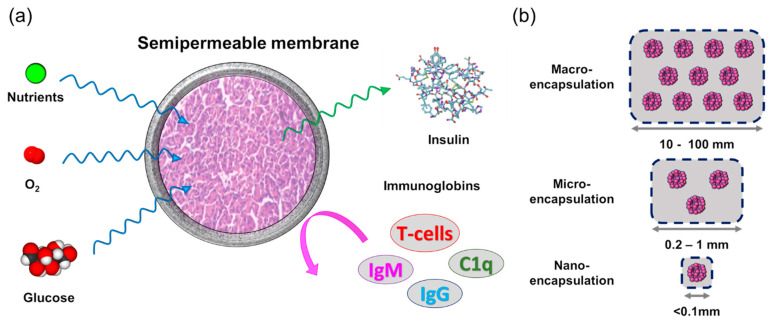
**(a**) Semipermeable membrane for bioartificial pancreas; (**b**) classification and dimension for each type of islet encapsulation devices.

As illustrated in Figure 16a, the capsule that encapsulates the islet cells is a semipermeable membrane which can protect the islet cells from immunoglobin while providing the necessary nutrients, oxygen and glucose to the cells. The smallest immunoglobin, IgG, has a MW of 150 kDa [202]. Hence, the optimal membrane MWCO (or pore size) to achieve these objectives is around 50-150 kDa (of course, solute MW alone cannot and should not be the sole factor that determines the selectivity).

Depending on the size and type of islet cells, there are three types of islet encapsulation: macroencapsulation (10–100 mm), microencapsulation (0.2–1 mm), and nanoencapsulation (<0.1 mm) (Figure 16b). Macroencapsulation devices can be further classified into two different types—intravascular and extravascular—depending on the implant position. The intravascular device consists of islet cells encapsulated in hollow fiber membranes, implanted directly into the patient’s cardiovascular system. The extravascular device is mostly implanted in the peritoneal cavity of the patient. 

There are various types of membrane materials applied for the intravascular devices (Table 6) such as polyacrylontrile-polyvinylchloride (PAN-PVC) copolymer, polycarbonate, EVAL fibers, poly-animo-urethane, nonwoven PTFE fabric and nylon microporous membrane to encapsulate the islet cells [203,204,205,206]. 

As for the extravascular devices, inorganic materials have been reported, such as silica [207], aluminum/aluminum oxide [208,209] and titanium/titanium oxide [209]. However, the use of inorganic membranes for BAP is still relatively new and has not been clinically proven. Polymeric membranes are more widely used to encapsulate the islet cells (Table 6). Current membrane permselectivity is poor; it must be improved to decrease the insulin-release time delay. 

## 3. Summary and Conclusions

Artificial organ (AO) technology has become an indispensable tool for the treatment of organ failures. As summarized in this review, there have been incredible strides in many of AO devices that employ membrane technology including lung, kidney, liver, and pancreas. Every organ has its own unique functions, and the membrane must be tailor-developed for each organ. It is important to reiterate that the key bottleneck in membrane-based AO technology is not the permselectivity of the membrane (although better performance is still desired), but the hemocompatibility of the membrane material at the blood–material interface. The strategy to achieve better hemocompatibility must also be tailor-developed for each organ, as the requirements for kidney and lungs, for example, can be drastically different. In addition, particularly for artificial lungs, effective module design to improve the mass transfer coefficient is the most important parameter. Additionally, the development of a hemocompatible superamphiphobic membrane could be an important breakthrough. Unfortunately, only the physical functions of human organs can be partly augmented with the membrane technology. Bioartificial organ technology, which incorporates live cells, has been improving to replace the biochemical aspects of failed organs. Future research must focus on developing better bioartificial organs, precisely controlling the selective permeation of solutes in and out of the membrane to suppress the unwanted immune response. Additionally, future AO research needs to improve the well-being and quality of life of organ failure patients, until bridge-to-recovery or bridge-to-transplantation. 

## Figures and Tables

**Table 1:** Comparison of key parameters of human lungs and oxygenator membranes.

Parameters	Lung	Oxygenator Membrane	Ref.
Exchange surface area	70 m^2^	1–3 m^2^	[34,35]
Surface characteristics	Hydrophilic	Hydrophobic	[34]
Membrane thickness	1–2 μm	50–100 μm	[34]
Gas permeability	High	Low	[34]
Blood contact time	<1 s	5–15 s	[34]
Type of gas	Air	Enriched air	[34]
O_2_ quantity added into blood	2 L/min	>470 mL/min	[35]
CO_2_ quantity removed from blood	1.6 L/min	>370 mL/min	[35]

**Table 2:** Mass transfer correlations for different blood oxygenator devices.

Type of Membrane	Blood Flow Pattern	Correlation for Blood Side Mass Transfer Coefficient	Ref.
PP/Hollow fibers	Across hollow fiber mat	$Sh=0.8\;Re^{0.59}\;Sc^{0.33}$	[38]
PP/Flat sheet	In thin channel	Sh=0.5 Gz	[38]
PP /Hollow fibers	Across a 1 to 6 perpendicular hollow fibers	$Sh=Re^{0.67}\;Sc^{0.33}\exp\left(3.26\;\varepsilon_{f}-4.27\right)$ $(\varepsilon_{f}$ is the void fraction)	[39]
PP /Hollow fibers	Across a parallel hollow fibers	$Sh=0.46\;Re^{0.76}\;Sc^{0.33}$	[36]
PP /Hollow fibers	Across a perpendicular hollow fibers	$Sh=0.14\;Re^{0.9}\;Sc^{0.33}$	[36]
PMP /Hollow fibers	Across a parallel hollow fibers	$Sh=0.39\;Re^{0.76}\;Sc^{0.33}$	[40]

**Table 3:** Generalized mass transfer correlations with parallel and transverse flow for hollow fiber blood oxygenator in square or staggered arrangement (with $\varepsilon_{f}$ is the void fraction of the bundle of hollow fibers) (equations obtained from [37]).

Flow Direction	Gas Type	Fiber Bundles Arrangement	
		Square	Staggered
Parallel flow	O_2_	$Sh=0.0180\varepsilon_{f}^{-8.7133\log\left(\varepsilon_{f}\right)-4.8654}$ $Re^{0.3050\varepsilon_{f}^{3.2197\log\left(\varepsilon_{f}\right)+0.9927}.}\;Sc^{0.33}$	$Sh=0.0584\varepsilon_{f}^{-0.751}\;$ $Re^{0.237\varepsilon_{f}^{-0.286}.}\;Sc^{0.33}$
	CO_2_	$Sh=0.3814\varepsilon_{f}^{-4.7273\log\left(\varepsilon_{f}\right)-2.6162}\;$ $Re^{0.3310\varepsilon_{f}^{3.6219\log\left(\varepsilon_{f}\right)+1.6150}.}\;Sc^{0.33}$	$Sh=0.6735\varepsilon_{f}^{-0.578}\;$ $Re^{0.2082\varepsilon_{f}^{-0.142}.}\;Sc^{0.33}$
Transverse flow	O_2_	$Sh=0.0892\varepsilon_{f}^{-0.864}\;$ $Re^{0.3288\varepsilon_{f}^{-0.051}.}\;Sc^{0.33}$	$Sh=0.1311\varepsilon_{f}^{-0.666}\;$ $Re^{0.3433\varepsilon_{f}^{-0.034}.}\;Sc^{0.33}$
	CO_2_	$Sh=0.3838\varepsilon_{f}^{-0.611}\;$ $Re^{0.2676\varepsilon_{f}^{-0.055}.}\;Sc^{0.33}$	$Sh=0.5216\varepsilon_{f}^{-0.505}\;$ $Re^{0.2547\varepsilon_{f}^{-0.130}.}\;Sc^{0.33}$

**Table 4:** The intrinsic gas permeability (barrer) of oxygen and carbon dioxide at 30 °C for dense polymeric films.

Polymer	P(O_2_)	P(CO_2_)	Ref.
Polypropylene (PP)	2.2	9.2	[51,52]
Poly 4-methyl pentene-1 (PMP)	32.3	92.6	[52]
Polydimethylsiloxane (PDMS)	605	3240	[52]
Teflon AF2400	1600	3900	[53]
Teflon AF1600	270	520	[53]
Hyflon AD80	67	150	[53]
Hyflon AD60	57	130	[53]
Natural rubber	23.3	153	[52]
Polyethylene (dens. 0.922)	6.9	28	[52]
Polytetrafluoroethylene (PTFE)	4.9	12.7	[52]
Neoprene	4	25.8	[52]
Polystyrene	2.63	10.5	[51,52]
Polycarbonate	1.4	8	[52]
Butyl rubber	1.3	5.18	[52]
Cellulose acetate	0.8	2.4	[52]
Polyvinyl chloride (unplasticized)	0.045	0.16	[52]
Nylon 6	0.038	0.16	[51,52]
Polyethylene terephthalate	0.035	0.17	[52]
Polyvinylidene chloride	0.0053	0.029	[52]
Polymethacrylonitrile	0.0012	0.0032	[52]
Polyacrylonitrile	0.0003	0.0018	[52]

Units: Barrer- cm^3^ (STP).cm/(sec.cm^2^.cmHg × 10^10^)

**Table 5:** Specifications of current commercial hollow fiber membrane blood oxygenator devices.

Device	Capiox FX	Affinity NT	Quadrox	Vision	Vital	Eurosets	Hilite	Hilite LT
**Company**	Terumo	Medtronic	Maquet	Gish Biomedical	NIPRO	Eurosets	Medos	Medos
**Membrane materials**	PP	PP	PP	PP	PP	PMP	PP	PMP
**Membrane area, m^2^**	0.5–2.5	2.5	1.8	2.45	2	0.69–1.81	0.39–1.9	0.32–1.9
**Priming volume, ml**	43–260	270	250	280	180	90–225	57–275	55–275
**Blood flow, L/min**	0.1–7	1–7	0.5–7	1–8	0.5–7	0.2–7	1–7	0.8–7
**O_2_ transfer rate, mL/min**	50–500	50–400	Max 425	400	_	90–350	_	100–550
**CO_2_ transfer rate, mL/min**	50–500	50–400	Max 320	200–500	_	70–300	_	75–350
**Coating materials**	X coating (nonheparin)	Balance biosurface (heparin-free)	SOFTLINE coating	GBS coating (nonleaching heparin coating)	_	Phosphorylcholine	Uncoated/ x.eed/ rheoparin	Uncoated/ rheoparin
	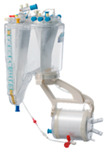	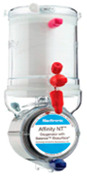	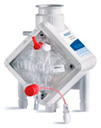	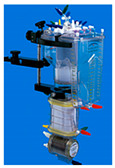	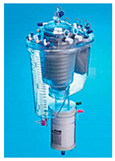	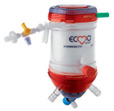	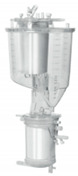	

**Table 6:** Polymeric membranes were applied for intra/extravascular microencapsulation devices.

Type of devices	Material	Islet source	Model for	Ref.
Intravascular	PAN-PVC	Rat, Monkey	Rat, Monkey	[203]
	Polycarbonate	Rat	Dog	[204]
	EVAL fibers	Porcine	Pig	[205]
	Poly-animo-urethane-coated			
	Nonwoven PTFE fabric			
	Nylon	Rabbit fetuses	Human	[206]
Extravascular	Nitrocellulose acetate	Mice	Mice	[210]
	2-Hydroxyethyl methacrylate	Rat, Rabbit	Rat	[211]
	Cellulose acetate	Human	Rat	[212]
	Acrylic copolymer	Rat	Mice	[213]
	Acrylonitrile (AN62)	Rat	Rat	[214]
	Polysulfone	Rat	In vitro	[215]

## Abbreviations

**Abbreviation**	**Full Name**
AI	Artificial intelligence
AN62	Acrylonitrile
AO	Artificial organ
BAK	Bioartificial kidney
BAL	Bioartificial liver
BAP	Bioartificial pancreas
BLSS	Bioartificial liver support system
CFD	Computational fluid dynamic
CIT	Clinical islet transplantation
CPB	Cardiopulmonary bypass system
CTA	Cellulose-triacetate
cTAL	Compliant thoracic artificial lung
ECMO	Extracorporeal membrane oxygenation
ELAP	Extracorporeal liver assist device
EVAL	Ethyl-vinyl-acetate copolymer
FPSA	Fractionated plasma separation and adsorption
GPU	Gas permeation unit
HD	Hemodialysis
HDF	Hemodiafiltration
HF	Hemofiltration
HF	Hollow fiber
HFP	Hexafluoropropylene
IVOX	Intravenacaval oxygenator and carbon dioxide removal device
MARS	Molecular adsorbent recycling system
MW	Molecular weight
MWCO	Molecular weight cut-off
NIPS	Nonsolvent induced phase separation
N-TIPS	Nonsolvent-thermally induced phase separation
P(VDF-CO-HFP)	Poly(vinylidene fluoride)-co-hexafluoropropylene
PAAL	Paracorporeal ambulatory assist lung
PAES	Polyarylethersulfones
PAN	Polyacrylontrile
PAN-PVC	Polyacrylontrile-polyvinylchloride
PC	Phosphorylcholine
PDMS	Polydimethylsiloxane
PES	Polyethersulfone
PMEA	Poly(2-methoxyethylacrylate)
PMMA	Polymethylmethacrylate
PMP	Poly-4-methylpentence
POSS	Polyhedral oligomeric silsesquioxanes
PP	Polypropylene
Psu	Polysulfone
PTFE	Polytetrafluoroethylene
PVDF	Poly(vinylidene fluoride)
PVP	Polyvinylpyrrolidone
RAD	Renal tubule assist device
SEPET	Selective plasma filtration therapy
SPAD	Single-pass albumin dialysis
TIPS	Thermally induced phase separation
WAK	Wearable artificial kidney
